# The Effect of Er,Cr:YSGG and Diode Laser Applications on Dental Implant Surfaces Contaminated with *Acinetobacter Baumannii* and *Pseudomonas Aeruginosa*

**DOI:** 10.3390/ma12132073

**Published:** 2019-06-27

**Authors:** Adel S. Alagl, Marwa Madi, Sumit Bedi, Faisal Al Onaizan, Zainab S. Al-Aql

**Affiliations:** 1 Department of Preventive Dental Sciences, College of Dentistry, Imam Abdulrahman Bin Faisal University, Dammam 31441, Saudi Arabia; 2 Department of Restorative Dental Sciences, College of Dentistry, Imam Abdulrahman Bin Faisal University, Dammam 31441, Saudi Arabia; 3 Dental Services, King Abdulaziz Medical City, National Guard Health Affairs, Jeddah 21423, Saudi Arabia

**Keywords:** dental implant, Er,Cr:YSGG, *Acinetobacter baumannii*, Pseudomonas aeruginosa, diode laser

## Abstract

Treatment of peri-implantitis through several implant surface decontamination techniques have been reported, however, some of them can negatively alter the implant surface or enhance more bacterial resistance. The aim of this in vitro study was to evaluate implant surface decontamination by means of Er,Cr:YSGG and diode lasers. Fifty micro-textured (MTX) dental implants were contaminated with *Acinetobacter baumannii* (*n* = 25) and with *Pseudomonas aeruginosa* (*n* = 25). All implants were then divided into five groups for the decontamination procedure. In group I (GI), decontamination was done with an Er,Cr:YSGG laser (2780 nm), while in group II (GII) decontamination was performed using photodynamic therapy (a 650 nm diode laser). In Group III (GIII) decontamination was performed with photodynamic therapy (an 808 nm diode laser), and in group IV (GIV) decontamination was performed with 0.12% chlorhexidine. Group V (GV) was the control group with no decontamination. After decontamination, colony forming units (CFU) were counted and implants were prepared for SEM analysis. A significant difference (*p* < 0.001) was observed for GI compared to the other groups, and also for GIV compared to both GII and GIII. The Er,Cr:YSGG laser (GI) showed the best results in decontaminating the implant surface. Chlorhexidine (GIV), proved to be better in decontaminating the implant surface than photodynamic therapy GII and diode laser GIII. No significant difference was found between group GII and GIII. The SEM analysis showed no significant change in the implant surface topography. The results of this study suggest that the Er,Cr:YSGG laser can be considered as an effective technique for reducing bacteria contamination on implant surfaces.

## 1. Literature Survey

Peri-implantitis is an inflammatory condition affecting the tissues surrounding a dental implant and is usually associated with progressive pocketing and bone loss [1]. Peri-implantitis continues to pose a serious challenge to the success of osseointegration of dental implants. The goals to be achieved during the surgical stage of implant treatment include plaque elimination, implant surface decontamination, tissue rejuvenation, and preservation of a healthy post-operative oral environment [2].

Numerous treatment approaches have been reported in the literature trying to achieve effective decontamination for dental implants [3,4,5,6,7]. Mechanical methods using ultrasonic scalers or Ti brushes, as well as chemical procedures using hydrogen peroxide or chlorhexidine have been reported [8,9,10]. However, some of these procedures impaired the implant surface topography, encourage bacterial resistance, or did not show re-osseointegration [11,12].

Recently lasers have been tested in the decontamination of dental implant surfaces and most commonly used lasers for this include soft lasers like CO_2_ [13], diode, and erbium lasers because of their ability to achieve hemostasis, ablation of calculus, and bactericidal action. On the contrary, higher-power lasers are expensive and can encourage an undesirable increase in temperature and alterations in the surface [14,15].

Erbium chromium-doped:yttrium, scandium, gallium, and garnet 2790 nm lasers (Er,Cr:YSGG) are highly absorbed by OH-ions compared to water molecules, and they have been used previously for removing calcified deposits, such as calculus, without altering the tooth/implant surfaces [16,17]. Strever et al. showed the effectiveness of Er,Cr:YSGG in decontaminating different implant surfaces from *Pseudomonas gingivalis* biofilm [18]. Additionally, previous in vitro study showed that decontaminating the implant surface using an Er,Cr:YSGG laser favors osteoblast attachment [19].

Photodynamic therapy (PDT) is the use of a diode laser to activate a specific photosensitizer agent to produce singlet oxygen which is toxic to the target [20]. Previous studies used PDT for implant decontamination without damaging the dental implant surfaces [9,21,22,23]. The inhibitory effect of the diode laser (808 nm) on bacteria/LPS adherent to titanium oxide was observed spectrophotometrically through decreases in nitrous oxide/nitrite production which is an indicator of macrophage activation [24].

However, there is still no consensus amongst researchers about the irradiation parameters which would enhance the efficacy of PDT or erbium lasers in achieving decontamination. 

*Acinetobacter baumannii* and *Pseudomonas aeruginosa* are Gram-negative bacteria that show multidrug-resistance and have been associated with multiple healthcare-associated infections [22]. Due to their direct involvement in serious nosocomial infections, *A. baumannii* and *P. aeruginosa* are of great concern for public health [25].

Thus, the purpose of this study was to investigate and compare the effect of Er,Cr:YSGG and diode lasers on the removal of *A. baumannii* and *P. aeruginosa* biofilm on dental implant surfaces. 

## 2. Material and Methodology

**Figure 1:**
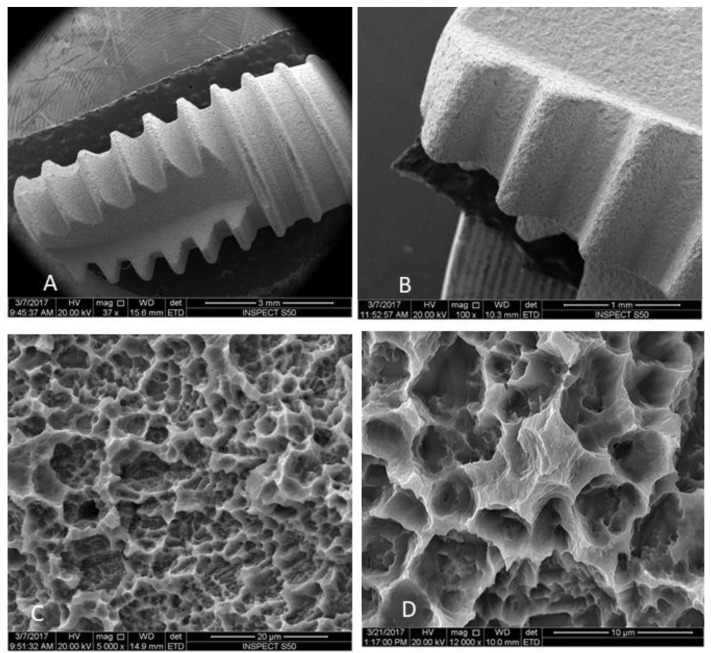
Scanning electron micrographs of MTX implants used in the current study (**A**,**B**) Higher magnification of the MTX surface before surface contamination: (**C**) 5000×; and (**D**) 12,000×.

Fifty micro-textured treatment implants (tapered screw-vent MTX, Zimmer Dental Inc., Carlsbad, CA, USA), 4.1 mm × 11.5 mm, rough surface grit blasted using hydroxyapatite (HA) were used (Figure 1). The implants have uniform surface of closely spaced micropits with an Sa value of 3.30 ± 0.22 µm. 

### 2.1. Manipulation

Fifty implants were divided into Groups A and B (*n* = 25). Group A was subdivided into five groups (*n* = 5) and all were contaminated with *A. baumannii*. Group B was subdivided into five groups (*n* = 5) and all were contaminated with *P. aeruginosa*. Two reference strains, *A. baumannii* (ATCC BAA-1710) and *P. aeruginosa* (ATCC 27853), were grown on Luria–Bertani media (LB) at 37 °C and overnight cultures were used [20,26]. A suspension with 3 × 10^5^ CFU/mL concentration was prepared by spectrophotometer. Implants were placed in 3 mL of bacterial suspension for 48 h for biofilm formation. After formation of the biofilms, unattached cells were removed by washing with sterile phosphate-buffered saline (PBS), placed in a new sterile Eppendorf tube, and randomly allocated to the different treatment groups.

**Figure 2:**
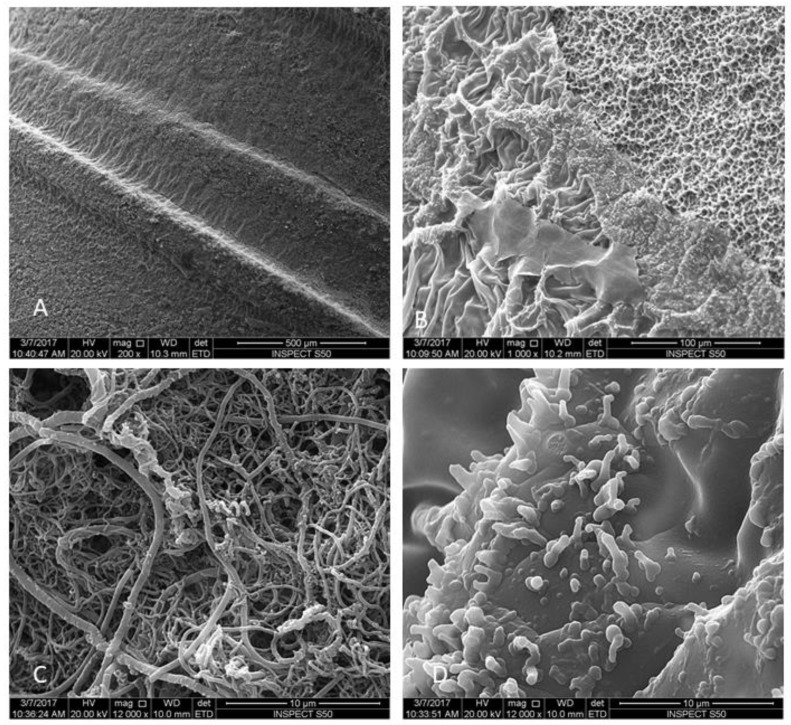
Scanning electron micrographs of MTX implants after bacterial contamination Group V. Low magnification showing the biofilm formation on implant threads: (**A**) 200×; and (**B**) 1000×. Higher magnification show biofilm colonization (48 h) of untreated implants. A multilayered *P. aeruginosa* biofilm was present on implant surfaces: (**C**) 12,000×. A multilayered *A. baumannii* biofilm was present on all implant surfaces: (**D**) 12,000×.

Decontamination procedures were performed, except for Group V, which serves as the negative control, and implants were prepared for SEM (Figure 2). 

### 2.2. Decontamination

A total of fifty MTX microtextured titanium implants were randomly divided into five groups (*n* = 10). Prior to decontamination methods, loosely adherent bacterial cells were removed by gently rinsing all the implants twice with 1.5 mL PBS (Table 1).

Group I: An erbium, chromium-doped yttrium, scandium, gallium, garnet (Er,Cr:YSGG laser) (Waterlase iPlus, Biolase, Irvine, CA, USA) device at a wavelength of 2.780 mm emitting an axial and radial laser beam was selected for laser irradiation. Irradiation was performed using short pulse H (60 µs) mode at a setting of 50% water and 50% air, 30 Hz, 1 W, and 120 J. 

The fiber tip used was an MZ8-6 mm (ZipTip) that was held perpendicular to the implant surface at a distance of 0.5 mm, using a custom-made mount and a scanning motion was used. The amount of time needed for irradiation was, on average, 60 s per implant surface. All treatments were performed by the same experienced operator. For operator’s safety, multi-wavelength protective eyewear was used and the cutting spray was directed toward the implant surface only.

Group II: The implants were immersed in 3 mL of the Toludine Blue O (Sigma, Poole, UK) at a concentration of 0.01% (mass per volume) for 5 min. Then laser irradiation was performed using a GaAlAs low-level diode laser (Lasercat 500, Medsolution, Radolfzell, Germany) at a wavelength of 650 nm and output power of 50 mW at a distance of 0.5 mm for 60 s per implant surface.

Group III: Treatment was completed with a diode laser (Lasercat 500, Medsolution, Radolfzell, Germany) at a wavelength of 808 nm. The exposed surface of the implant was irradiated in contact mode. The laser tip was positioned at a distance of 0.5 mm at one point, then at another point, and so on. Sequentially, until the irradiation time reached 60 s per implant surface.

Group IV: The implants were immersed in in 3 mL of 0.12% chlorhexidine solution for 120 s. 

Group V: No decontamination method was performed. 

After decontamination, all the samples were gently irrigated with PBS to avoid transportation of any chemical residue to the culture media, which can alter colony growth. All groups were similarly irrigated with PBS for the purpose of standardization, by the same calibrated operator.

### 2.3. Analysis of Decontamination

Quantitative analysis of decontamination was done by inoculation of microbes in a culture media followed by counting colony forming units (CFU) using a stereoscopic microscope at 10× magnification. After decontamination of different groups, the implants were placed in sterile centrifuge tubes and agitated for 30 s. After serial dilutions, 1 mL of each dilute was cultured on Brucella agar [27]. The plates were incubated at 37 °C for seven days. The number of bacteria per milliliter was calculated manually by blind expert examiner using a “click–counter” and a pen.

### 2.4. SEM Evaluation

All samples were subjected to SEM evaluation to study the topographical changes in implant surface and remaining bacterial biofilm. The implants were placed in fixative solution (4% paraformaldehyde, 2% glutaraldehyde in 0.1 M sodium cacodylate (NaCac)) buffer, pH 7.4, overnight and post-fixed in 2% osmium tetroxide in NaCac buffer, dehydrated through a graded ethanol series (25–100%), and critical-point dried using CO_2_ (Samdri790, Tousimis, Inc., Rockville, MD, USA). The dried implants were mounted on aluminum stubs with carbon adhesive tabs, electrically grounded with colloidal graphite, and sputter-coated for 6 min with gold-palladium (Anatech Hummer™6.2, Union City, CA, USA). The implants were imaged by a scanning electron microscope SEM (FEI, Inspect S50, Berno, Czech Republic), which was operated at 20 kV and electronic images were then captured. Four images were then recorded from each implant at the apical three threads using 1000×, 6000×, and 12,000× magnification. Representative images of each treatment were selected for presentation.

### 2.5. Statistical Analysis

IBM SPSS for Windows version 22.0 (IBM Corp., Armonk, NY, USA) was used for statistical analysis. Normality was assessed using the Kolmogorov–Smirnov test. Due to the non-normal distribution, the Kruskal–Wallis test was used to assess difference among groups followed by pairwise comparisons among groups. Significance was set at 5%. 

## 3. Results

**Figure 3:**
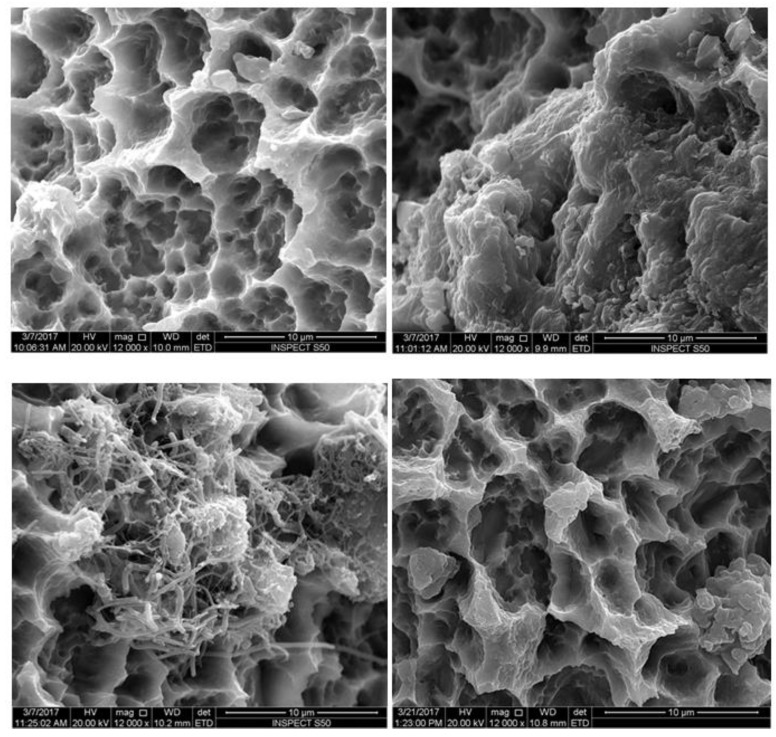
Scanning electron micrographs of MTX implants contaminated with *A. baumannii*. Gp I: Er,Cr:YSGG-treated groups, no bacteria were visible allowing the original titanium surface to be observed (**A**). Gp II: PDT-treated group showing a multilayered *A. baumannii* biofilm on the implant surface (**B**), Gp III: Diode laser (808 nm)-treated group showing bacterial colonization on the implant surface (**C**), Gp IV: The 0.12% chlorhexidine-treated group showing few bacteria colonization (white arrow) (**D**). Magnification: 12,000×.

**Figure 4:**
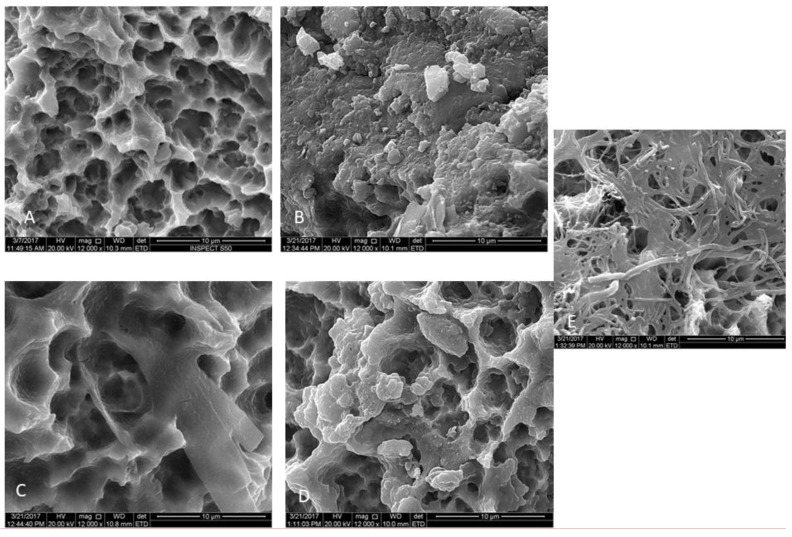
Scanning electron micrographs of MTX implants contaminated with *P. aeruginosa*. Gp I: Er,Cr:YSGG-treated groups; no bacteria were visible, allowing the original titanium surface to be observed (**A**). Gp II: PDT-treated group showing a multilayered *P. aeruginosa* biofilm on the implant surface (**B**), Gp III: Diode laser (808 nm)-treated group showing bacterial colonization on the implant surface (**C**), Gp IV: The 0.12% chlorhexidine treated group showing few bacteria colonization (white arrow) (**D**). Gp V: The no decontamination group showing a massive multilayered bacterial biofilm covering the implant surface (**E**). Magnification: 12,000×.

The treatment with GI (Er,Cr:YSGG) showed a significantly fewer viable cells compared to other treatment groups, as well as the untreated group (*p* < 0.0001, Table 2, Figure 3 and Figure 4). Next to Group GI, GIV had significantly lower levels of microbes after decontamination compared to groups GII, GIII, and GV (Figure 3 and Figure 4)

### SEM Analysis

The SEM images showed a rough and irregular honeycomb surface, which is the characteristic surface for MTX implants. Most of *A. baumannii* and *P. aeruginosa* were ablated from the implant surface in group I (Er,Cr:YSGG laser). The treatment did not alter the morphological appearance of the implant surface. The honeycomb appearance was not observed in both groups II and III, however, a dense bacterial colonization covered the implant surface. The attached bacteria were distributed with different patterns forming chains and clusters. Residual bacterial cells remaining could be observed in Group IV (CHX) in pits and fissures, as well as some on smooth surfaces, but with sparse distribution compared to groups II and III. 

## 4. Discussion

In recent decades, several researchers have proved the association of periodontal microbes/disease and systemic diseases. Previous studies have detected non-oral pathogenic microbes in the saliva and/or biofilms in the oral cavity and indicated that the oral cavity may be a reservoir for medically-important pathogens [28,29,30]. In addition to recognized periodontal pathogens, studies have shown the presence of species such as *Acinetobacter* spp. and *P. aeruginosa*, and concluded that these non-oral pathogens may also play a role in the etiopathogenesis of periodontal diseases [28,31]. Sousa et al. investigated the bacterial microbiome in periodontal and peri-implant biofilms deriving from aggressive periodontitis patients and found that *Acinetobacter* were unique to peri-implant sites [32]. Albertini et al. and Zhang et al. [33,34] reported higher proportions of *Pseudomonas* species in peri-implant sites [34]. Other authors have also reported associations between these species and sites and/or subjects with periodontal disease, particularly in HIV-infected patients [29]. Likewise, Colombo et al. suggested that *P. aeruginosa* in epithelial tissues might be of potential use as a diagnostic tool for periodontitis [35]. The existence of cell-surface lipopolysaccharides, the capacity to yield biofilms, and resistance to multiple drugs may favor colonization in the periodontal tissues. Due to these reported features, *Acinetobacter* spp. and *P. aeruginosa* were selected for this study.

Biofilm formation is a special characteristic of *A. baumannii* and *P. aeruginosa*, which make them of the most important and most dangerous nosocomial pathogens [36]. The biofilm provides them the ability to persist and survive on various environmental surfaces under severe conditions for long duration with high resistance to antibiotics and dehydration [37,38]. Thus, for hospitalized and immunocompromised patients these bacteria can endure on medical devices and in host tissues causing serious complications [36].

With the increasing success of rehabilitation with dental implants, more cases of failure due to peri-implantitis also have reported in recent times. Thus, management of peri-implantitis poses a frequent challenge to dentists. With the purpose of disinfecting the implant surface, various treatment modalities have been suggested, but none of them have shown satisfactory results until now. Photodynamic therapy appears to be a possible alternative to minimize bacteria on implant surfaces. The advantages of laser and PDT in dentistry are well documented in the literature and, recently, in dental implantology and as a co-adjuvant treatment for peri-implantitis, its use has been supported. Thus, the purpose of this study was analyze the effect of Er,Cr:YSGG and diode lasers on the removal of *A. baumannii* and *P. aeruginosa* biofilm on dental implant surfaces.

In contrast to previous studies [18,19,24,39] that used Ti discs, we used commercially available implants to closely simulate the clinical situations regarding the complex implant topography. Strever et al. showed that a bacterial biofilm on a Ti disc was easily removed even by water spray [18]. In this study, the surface roughness was not changed in any of the study groups. Surface roughness was shown to be an important factor to achieve osseointegration. Altering the Sa value of the implant will adversely influence the re-osseointegration process. Therefore, maintaininig the Sa of the implant after the decontamination method is an important goal that should be achieved for future re-osseointegration.

Increasing the laser duration or power setting can lead to complete elimination of residual bacterial biofilm, however, this would have led to changing the surface topography of the implants. The settings used in our study were selected to be within the range of the clinically used ones. 

Similar results were observed when using the Er,Cr:YSGG laser for *P. gingivalis* ablation, causing a 95% removal of biofilm without altering the surface roughness or changing the surface temperature of titanium discs [18].

The results of this study corroborate with previous studies reported in the literature. It was observed that more bacteria were found in Groups GII and GIII, in comparison with the Er,Cr:YSGG (GI) and chlorhexidine (G IV) groups. The results suggest that Er,Cr:YSGG can successfully decontaminate the implant surfaces and that low-level lasers are less effective (*p* < 0.001) as compared to PDT. Photodynamic therapy can be considered a co-adjuvant in management of peri-implantitis. Future studies should focus to test the different laser parameters, timing and other variables in order to design an ideal protocol for the usage of an Er,Cr:YSGG laser for the treatment of peri-implantitis patients.

A limitation of this study is the use of a single Er,Cr:YSGG laser setting, so trying different setting might be needed in order to determine the optimal setting limit that can be used without surface damage. Another limitation is using a single implant surface, so using different implant surfaces to examine the efficacy of the recommended laser settings on them is also recommended. 

## 5. Conclusions

Within the limitations of this study, an Er,Cr:YSGG laser with 1 Watt of power, for 30 s showed complete elimination of *A. baumannii* and *P. aeruginosa* biofilm on the implant surface. The Er,Cr:YSGG laser is an effective alternative treatment modality for decontamination of dental implant surfaces without damaging the surface topography.

## Figures and Tables

**Table 1:** Experimental groups and treatment modalities used in the study.

Groups	Group A Implants Contaminated with *A. Baumannii*	Group B Implants Contaminated with *P. Aeruginosa*	Decontamination Method	Watt	Time
Group I	5 implants	5 implants	Er,Cr:YSGG (2780 nm H mode)	1 W, 30 Hz, Air and water 50% contact mode 2 mm (120 J) then 3 mL PBS (twice)	60 s
Group II	5 implants	5 implants	650 Diode + TBO 1mg/mL (5 min in dark)	50 mW contact mode 2 mm (60 J) then 3 mL PBS (twice)	60 s
Group III	5 implants	5 implants	808 Diode	1 W contact mode 2 mm (120 J) then 3 mL PBS (twice)	60 s
Group IV	5 implants	5 implants	CHX 0.12%	then washed with 3 mL PBS (twice)	120 s
Group V	5 implants	5 implants	Bacterial contamination only		

**Table 2:** Colony forming units (CFU) for the tested bacteria in relation to the different decontamination method.

Study Groups	*Acinetobacter Baumannii*	*Pseudomonas Aeruginosa*
Group I Er,Cr:YSGG (2790 nm)	1.2 ± 1.3 ^a^	1.4 ± 1.3 ^a^
Gp II PDT	9176 ± 401 ^b^	7673 ± 307 ^b^
Gp III 808	4360 ± 421 ^c^	8156 ± 552 ^b^
Gp IV CHX	1536 ± 382 ^d^	1464 ± 207 ^c^
GpV cont	10,880 ± 563 ^e^	12,362 ± 481 ^d^
*p* value	<0.0001 *	<0.0001 *

^a,b,c,d,e^: different letters denote statistically significant differences, *: Statistically significant at *p* < 0.05; ^a^: between GI and all other 4 groups regarding both *A. b* and *P. a* count; ^b^: between GII and all other 4 groups regarding *A. b* count. No significant difference was observed between GII and GIII regarding *P. a* count.; ^c^: between GIII and the other four groups regarding *A. b* count and between GIV and the other four groups regarding *P. a* count.; ^d^: between GIV and all other 4 groups regarding *A. b* count and between GV and the other four groups regarding *P. a* count.; ^e^: between GV and all other 4 groups regarding *A. b* count.

## References

[1] Lindhe J., Meyle J. (2008). Peri-implant diseases: Consensus Report of the Sixth European Workshop on Periodontology. J. Clin. Periodontol..

[2] Esposito M., Grusovin M.G., Coulthard P., Worthington H.V. (2008). The efficacy of interventions to treat peri-implantitis: A Cochrane systematic review of randomised controlled clinical trials. Eur. J. Oral Implantol..

[3] Koo K.-T., Khoury F., Keeve P.L., Schwarz F., Ramanauskaite A., Sculean A., Romanos G. (2019). Implant Surface Decontamination by Surgical Treatment of Periimplantitis. Implant Dent..

[4] Toma S., Behets C., Brecx M.C., Lasserre J.F. (2018). In Vitro Comparison of the Efficacy of Peri-Implantitis Treatments on the Removal and Recolonization of Streptococcus gordonii Biofilm on Titanium Disks. Masteries.

[5] Louropoulou A., Slot D.E., Van der Weijden F. (2014). The effects of mechanical instruments on contaminated titanium dental implant surfaces: A systematic review. Clin. Oral Implants Res..

[6] Al-Hashedi A.A., Laurenti M., Abdallah M.-N., Albuquerque R.F., Tamimi F. (2016). Electrochemical Treatment of Contaminated Titanium Surfaces in Vitro: An Approach for Implant Surface Decontamination. ACS Biomater. Sci. Eng..

[7] Al-Hashedi A.A., Laurenti M., Benhamou V., Tamimi F. (2017). Decontamination of titanium implants using physical methods. Clin. Oral Implants Res..

[8] Alotaibi M., Moran G., Grufferty B., Renvert S., Polyzois I. (2019). The effect of a decontamination protocol on contaminated titanium dental implant surfaces with different surface topography in edentulous patients. Acta Odontol. Scand..

[9] Htet M., Madi M., Zakaria O., Miyahara T., Xin W., Lin Z., Aoki K., Kasugai S., Zakaria O. (2016). Decontamination of Anodized Implant Surface with Different Modalities for Peri-Implantitis Treatment: Lasers and Mechanical Debridement with Citric Acid. J. Periodontol..

[10] Madi M., Htet M., Zakaria O., Alagl A., Kasugai S. (2018). Re-osseointegration of dental implants after periimplantitis treatments: A systematic review. Implant Dent..

[11] Louropoulou A., Slot D.E., Van der Weijden F.A. (2012). Titanium surface alterations following the use of different mechanical instruments: A systematic review. Clin. Oral Implants Res..

[12] Parlar A., Bosshardt D.D., Çetiner D., Schafroth D., Ünsal B., Haytaç C., Lang N.P. (2009). Effects of decontamination and implant surface characteristics on re-osseointegration following treatment of peri-implantitis. Clin. Oral Implants Res..

[13] Stübinger S., Henke J., Donath K., Deppe H. (2005). Bone regeneration after peri-implant care with the CO_2_ laser: A fluorescence microscopy study. Int. J. Oral Maxillofac. Implant..

[14] Marotti J., Geraldo-Martins V.R., Bello-Silva M.S., de Paula Eduardo C., Apel C., Gutknecht N. (2010). Influence of etching with erbium, chromium:yttrium-scandium-gallium-garnet laser on microleakage of class V restoration. Lasers Med. Sci..

[15] Kreisler M., Al Haj H., D’Hoedt B. (2003). Temperature changes induced by 809-nm GaAlAs laser at the implant-bone interface during simulated surface decontamination. Clin. Oral Implants Res..

[16] Ting C.-C., Fukuda M., Watanabe T., Aoki T., Sanaoka A., Noguchi T. (2007). Effects of Er,Cr:YSGG Laser Irradiation on the Root Surface: Morphologic Analysis and Efficiency of Calculus Removal. J. Periodontol..

[17] Schwarz F., Nuesry E., Bieling K., Herten M., Becker J. (2006). Influence of an Erbium, Chromium-Doped Yttrium, Scandium, Gallium, and Garnet (Er,Cr:YSGG) Laser on the Reestablishment of the Biocompatibility of Contaminated Titanium Implant Surfaces. J. Periodontol..

[18] Strever J.M., Lee J., Ealick W., Peacock M., Shelby D., Susin C., Mettenberg D., El-Awady A., Rueggeberg F., Cutler C.W. (2017). Erbium, Chromium:Yttrium-Scandium-Gallium-Garnet Laser Effectively Ablates Single-Species Biofilms on Titanium Disks Without Detectable Surface Damage. J. Periodontol..

[19] Romanos G., Crespi R., Barone A., Covani U. (2006). Osteoblast attachment on titanium disks after laser irradiation. Int. J. Oral Maxillofac. Implant..

[20] Takasaki A.A., Aoki A., Mizutani K., Schwarz F., Sculean A., Wang C.Y., Koshy G., Romanos G., Ishikawa I., Izumi Y. (2000). Application of antimicrobial photodynamic therapy in periodontal and peri-implant diseases. Periodontology.

[21] Romanos G.E., Gutknecht N., Dieter S., Schwarz F., Crespi R., Sculean A. (2009). Laser wavelengths and oral implantology. Lasers Med Sci..

[22] Tacconelli E., Cataldo M., Dancer S., De Angelis G., Falcone M., Frank U., Kahlmeter G., Pan A., Petrosillo N., Rodríguez-Baño J. (2014). ESCMID guidelines for the management of the infection control measures to reduce transmission of multidrug-resistant Gram-negative bacteria in hospitalized patients. Clin. Microbiol. Infect..

[23] Marotti J., Tortamano P., Cai S., Ribeiro M.S., Franco J.E., de Campos T.T. (2013). Decontamination of dental implant surfaces by means of photodynamic therapy. Lasers Med Sci..

[24] Giannelli M., Landini G., Materassi F., Chellini F., Antonelli A., Tani A., Zecchi-Orlandini S., Rossolini G.M., Bani D. (2016). The effects of diode laser on Staphylococcus aureus biofilm and Escherichia coli lipopolysaccharide adherent to titanium oxide surface of dental implants. An in vitro study. Lasers Med. Sci..

[25] Ušjak D., Ivković B., Božić D.D., Bošković L., Milenković M. (2019). Antimicrobial activity of novel chalcones and modulation of virulence factors in hospital strains of Acinetobacter baumannii and Pseudomonas aeruginosa. Microb. Pathog..

[26] Moley J.P., McGrath M.S., Granger J.F., Stoodley P., Dusane D.H. (2018). Reduction in Pseudomonas aeruginosa and Staphylococcus aureus biofilms from implant materials in a diffusion dominated environment. J. Orthop. Res..

[27] Miles A.A., Misra S.S., Irwin J.O. (1938). The estimation of the bactericidal power of the blood. J. Hyg..

[28] Souto R., Silva-Boghossian C.M., Colombo A.P.V. (2014). Prevalence of Pseudomonas aeruginosa and Acinetobacter spp. in subgingival biofilm and saliva of subjects with chronic periodontal infection. Braz. J. Microbiol..

[29] Gonçalves L.S., Souto R., Colombo A.P.V. (2009). Detection of Helicobacter pylori, Enterococcus faecalis, and Pseudomonas aeruginosa in the subgingival biofilm of HIV-infected subjects undergoing HAART with chronic periodontitis. Eur. J. Clin. Microbiol. Infect. Dis..

[30] Zuanazzi D., Souto R., Mattos M.B.A., Zuanazzi M.R., Tura B.R., Sansone C., Colombo A.P.V. (2010). Prevalence of potential bacterial respiratory pathogens in the oral cavity of hospitalised individuals. Arch. Oral Boil..

[31] Da Silva-Boghossian C.M., Souto R.M.D., Luiz R.R., Colombo A.P.V. (2011). Association of red complex, A. actinomycetemcomitans and non-oral bacteria with periodontal diseases. Arch. Oral Boil..

[32] Sousa V., Nibali L., Spratt D., Dopico J., Mardas N., Petrie A., Donos N. (2017). Peri-implant and periodontal microbiome diversity in aggressive periodontitis patients: A pilot study. Clin. Oral Implants Res..

[33] Zhuang L.F., Watt R.M., Mattheos N., Si M.S., Lai H.C., Lang N.P. (2016). Periodontal and peri-implant microbiota in patients with healthy and inflamed periodontal and peri-implant tissues. Clin. Oral Implants Res..

[34] Albertini M., Lopez-Cerero L., O’Sullivan M.G., Chereguini C.F., Ballesta S., Rios V., Herrero-Climent M., Bullon P. (2015). Assessment of periodontal and opportunistic flora in patients with peri-implantitis. Clin. Oral Implants Res..

[35] Colombo A.V., Barbosa G.M., Higashi D., Di Micheli G., Rodrigues P.H., Simionato M.R.L. (2013). Quantitative detection of Staphylococcus aureus, Enterococcus faecalis and Pseudomonas aeruginosa in human oral epithelial cells from subjects with periodontitis and periodontal health. J. Med Microbiol..

[36] Lee K., Yoon S.S. (2017). Pseudomonas aeruginosa biofilm, a programmed bacterial life for fitness. J. Microbiol. Biotechnol..

[37] Marti S., Soto S.M., Cisneros J., Pachón J., Pascual A., McQueary C., Actis L., Vilá J., Rodríguez-Baño J., Martínez-Martínez L. (2008). Biofilm formation in Acinetobacter baumannii: Associated features and clinical implications. Clin. Microbiol. Infect..

[38] Bogdan M., Drenjancevic D., Harsanji Drenjancevic I., Bedenic B., Zujic Atalic V., Talapko J., Vukovic D. (2018). In vitro effect of subminimal inhibitory concentrations of antibiotics on the biofilm formation ability of Acinetobacter baumannii clinical isolates. J. Chemother. (Florence, Italy).

[39] Saffarpour A., Nozari A., Fekrazad R., Saffarpour A., Heibati M.N., Iranparvar K. (2018). Microstructural Evaluation of Contaminated Implant Surface Treated by Laser, Photodynamic Therapy, and Chlorhexidine 2 percent. Int. J. Oral Maxillofac. Implants.

